# Navigating ADHD in Higher Education: Evaluating Psychosocial Interventions for Student Self-Esteem, Well-Being, and Quality of Life

**DOI:** 10.1192/bjo.2024.106

**Published:** 2024-08-01

**Authors:** Aminah Ali, Karen Collier, Tosin Mayomi

**Affiliations:** 1 Warwick Medical School, Coventry, United Kingdom; 2 Coventry and Warwickshire Partnership Trust, Coventry, United Kingdom

## Abstract

**Aims:**

Several studies have shown that individuals diagnosed with Attention Deficit Hyperactivity Disorder (ADHD) have difficulties in maintaining their psychological well-being and are at risk of negative impacts on their psychological health during higher education. Consequently, it is crucial to introduce targeted interventions to promote self-esteem, quality of life, and overall well-being to minimise potential adverse outcomes. For this reason, the main objective of this systematic literature review was to identify and evaluate studies on the target population that explored the effects of psychosocial interventions on dimensions of psychological well-being, such as self-esteem, well-being, or quality of life.

**Methods:**

A systematic literature review was conducted following the PICO approach and PRISMA guidelines. The electronic databases – MEDLINE, PsycINFO, Web of Science, PubMed, COCHRANE Central and Education Research Complete were searched for English-language studies published between 2013–2023 on interventions conducted in university-level ADHD students that impacted their psychological well-being. Exclusion criteria encompassed studies focusing on comorbid clinical diagnoses such as anxiety or depression outcomes and pharmacological interventions. Nine studies that met the inclusion criteria were identified.

**Results:**

Cognitive-behavioural therapy (CBT), interpersonal group therapy, and coaching emerged as interventions with the strongest evidence base for improving psychological well-being in university students with ADHD. The ACCESS (Accessing Campus Connections and Empowering Student Success) intervention, a CBT and mentoring programme, demonstrated increased well-being over time (p = 0.001, d = 0.45). Interpersonal group therapy yielded significant improvements in global self-esteem (p = 0.001, η2 = 0.12), with a significant difference from the control group (p = 0.01, η2 = 0.07), while the coaching intervention revealed significantly higher well-being scores in participants compared with the control (p = 0.05, R2 = 0.11).

**Conclusion:**

This systematic review found psychosocial interventions focussing on CBT, interpersonal group therapy, and coaching were effective in improving the psychological well-being of university students with ADHD. Future intervention studies should establish a specific ADHD-focused CBT approach and have more extended follow-up periods to understand long-term effectiveness. This review also identifies priority areas for additional research.

